# The Clinical Significance of Colon Polyp Pathology in Nonalcoholic Fatty Liver Disease (NAFLD) and Its Impact on Screening Colonoscopy in Daily Practice

**DOI:** 10.1155/2020/6676294

**Published:** 2020-12-10

**Authors:** Cosmas Rinaldi A. Lesmana, Levina S. Pakasi, Aru W. Sudoyo, Ening Krisnuhoni, Laurentius A. Lesmana

**Affiliations:** ^1^Department of Internal Medicine, Hepatobiliary Division, Dr. Cipto Mangunkusumo National General Hospital, Medical Faculty, University of Indonesia, Jakarta, Indonesia; ^2^Digestive Disease and GI Oncology Centre, Medistra Hospital, Jakarta, Indonesia; ^3^Department of Internal Medicine, Hematology and Oncology Division, Dr. Cipto Mangunkusumo National General Hospital, Medical Faculty, University of Indonesia, Jakarta, Indonesia; ^4^Department of Pathology Anatomy, Dr. Cipto Mangunkusumo National General Hospital, Medical Faculty, University of Indonesia, Jakarta, Indonesia

## Abstract

**Aim:**

Nonalcoholic fatty liver disease (NAFLD) has been known as a risk for the presence of colon polyp and CRC development. This study was aimed to find out the clinical significance of colon polyps' pathology among NAFLD patients.

**Method:**

A retrospective database study was done in patients who underwent elective colonoscopy within one-year period in a referral private hospital, Jakarta. Subjects were adult patients who also had documented abdominal ultrasound (US). The association between NAFLD and colonic polyp was analyzed using Chi-square test with odds ratio (OR) and its corresponding 95% confidence interval (CI).

**Results:**

A total of 138 adult patients were enrolled; 68 (51.1%) were men. Patients' mean age was 56.8 ± 15.3 years old. Colon polyps were found in 49 (35.5%) cases; the most common histopathology was adenoma (42.9%). NAFLD was found in 68 (49.3%) of patients. Colon polyps were found to be more among patients with NAFLD than in those without NAFLD (44.1% vs. 27.1%; OR: 2.119; 95% CI: 1.040–4.318). Colon polyps were found in 30 (44.1%) NAFLD patients, where 18 (26.5%) patients had adenomatous polyp, and from this subset of patients with adenomatous polyp, 6 (8.8%) patients had mild dysplasia, 8 (11.8%) had moderate dysplasia, 1 (1.5%) had severe dysplasia, and 3 (4.4%) had adenocarcinoma.

**Conclusions:**

NAFLD is associated with increased risk of any colon polyp, regardless of the histopathological type, compared with patients without NAFLD. This finding implies the necessity to perform screening colonoscopy in patients with NAFLD in the future.

## 1. Background

Colorectal cancer (CRC) is still one of the most common cancers in the Western and Asian countries. Until now, the general recommendation for CRC screening is still based on invasive diagnostic tests, such as colonoscopy, and noninvasive tests, such as Asia-Pacific colorectal screening score (APCS) and fecal immunochemical test (FIT) [1, 2]. However, the patient's reluctance for invasive screening tests, different subgroups of patient's diversity, different screening program policies in every country, especially in developed and developing Asian countries, cost difference, and availability of diagnostic tools make a different strategy approach in the real clinical practice.

Nonalcoholic fatty liver disease (NAFLD) has been known to be associated with extrahepatic causes of death, including CRC [3]. Early studies in Asia have observed an increase of CRC risk in patients with ultrasound-diagnosed NAFLD compared with controls [4, 5]. The presence of NAFLD was found as an independent risk factor for colorectal adenomatous polyps in asymptomatic subjects who underwent routine colonoscopy [6, 7]. A similar finding was also reported in Western population [8]. A systematic review involving 6263 asymptomatic subjects undergoing screening colonoscopy confirmed the association between colorectal adenoma and NAFLD [9].

A large prospective cross-sectional study in a referral private hospital in Indonesia showed that NAFLD is a common finding in unselected adult patients who underwent routine medical check-up. Several risk factors were confirmed as independent risk factors for NAFLD, that is, obesity, male gender, age of more than 35 years, high triglyceride, low high-density lipoprotein (HDL) cholesterol, and high serum alanine aminotransferase levels [10]. Notwithstanding, retrospective data analysis study of a large sample size in the same private hospital for colon polyps or cancer detection rate found almost similar important risk factors, that is, older age (≥50 years old) and male gender [11].

However, until now, there is no consensus about how the CRC screening should be done in NAFLD population. Therefore, considering the increased prevalence of NAFLD in younger age, possible genetic and environment differences, different CRC molecular pathways, and different conclusion of pathologist's specimen evaluation, the author would like to highlight this issue based on the pathology finding of colon polyp in NAFLD patients.

## 2. Method

### 2.1. Study Design and Subjects

A retrospective database study was done in unselected patients who underwent elective colonoscopy within one-year period in Medistra Hospital, Jakarta. Data was collected on the patients' demography and colonoscopy findings including the presence of hemorrhoids, polyps, diverticula, inflammation, or tumor mass. Patients were included if they also had a transabdominal ultrasound study record, as it is the preferred first-line diagnostic imaging procedure for imaging screening of NAFLD [12]. Patients with evidence of hepatitis virus infection, autoimmune hepatitis, significant alcohol consumption, history of any other cancers, or familial adenomatous polyposis (FAP) were excluded.

### 2.2. Colonoscopy Procedure

Following full bowel preparation, a conventional colonoscopy procedure was performed in all patients using adult high-definition video fiber optic colonoscopy with an auxiliary water jet (CFQ160AL, Olympus, USA). Polyps were removed by snare-loop polypectomy at the bottom of the stalk or by biopsy forceps. Flat or sessile polyps were removed by performing a submucosal lifting technique with the injection of saline solution into the submucosal layer. Polypectomy was not performed in patients with known absolute contraindications such as anticoagulant therapy and bleeding disorders.

### 2.3. Histopathological Diagnosis

Biopsy specimens were evaluated and classified according to the World Health Organization (WHO) Classification by two board-certified pathologists who were blinded to clinical data. High-grade dysplasia or in situ carcinoma is defined by the considerable loss of nuclear polarity with irregular glandular architecture with no involvement beyond the muscularis mucosa. Submucosal invasive early colorectal cancer was defined as malignant lesions that invade the submucous layer, while cancer was defined as invasion of malignant cells beyond the muscularis mucosa.

### 2.4. Statistical Analysis

Patients' demography was presented descriptively. Categorical data were compared using the Chi-square test. A *p* value of less than 0.05 was considered significant. Risk for polyp development in patients with NAFLD compared with patients without NAFLD was expressed as odds ratio (OR) and its corresponding 95% confidence interval (CI). Median difference was assessed using Mann–Whitney *U* test for skewed data. Statistical analysis was done using the SPSS software version 19.0 (SPSS Inc., Chicago, Illinois, USA).

## 3. Results

Four hundred and fourteen patients underwent elective colonoscopy during the study period; however, only 138 patients had documented of transabdominal ultrasound results. Seventy-two patients (52.2%) were men and their mean age was 56.8 ± 15.3 years old. NAFLD was diagnosed in 68 (49.3%) of patients. Colon polyps were found in 49 (35.5%) patients (Table 1). The most common histopathology was adenoma and mostly having a mild dysplasia (Table 2).

Colon polyps were found among patients with NAFLD more than those without NAFLD (44.1% vs. 27.1%; *p*=0.037). The presence of NAFLD was associated with 2.1 times increased risk of the presence of colon polyp (Table 3). The median age of patients with polyp tended to be higher than that of patients without polyp (59 vs. 56 years;*p*=0.639, Mann–Whitney *U* test). Colon polyp was found in 30 (44.1%) NAFLD patients, where 18 (26.5%) patients had adenomatous polyp, and from this subset of patients with adenomatous polyp, 6 (8.8%) patients had mild dysplasia, 8 (11.8%) had moderate dysplasia, 1 (1.5%) had severe dysplasia, and 3 (4.4%) had adenocarcinoma (Table 4). Based on the polyps' location, 10 (33.3%) patients had a right-sided polyp, and 20 (66.7%) patients had a left-sided polyp (Table 5).

Based on dysplasia's type, there was no statistically significant difference between the presence of mild and moderate-to-severe grade of dysplasia in NAFLD patients (Table 6).

## 4. Discussion

The impact of colon polyps' pathology in NAFLD patients as a prevention for CRC development has not been well documented. Our current study in Indonesia, which represents the biggest Southeast Asian country, is the first report showing an association between NAFLD and colorectal polyp. Generally, there are two responsible important factors known as the cause of the CRC development, such as genetic predisposition (FAP and HNPCC) and environmental factors; however, sporadic CRC has been noted as the most common pathway in CRC development [13]. Sporadic pathway is usually based on chromosomal instability (CIN) or microsatellite instability (MSI). This pathway contributes to CRC development in patients with a median age of 70–75 years old. However, molecular CRC study in Indonesia showed that sporadic CRC was found in younger patients (40 years old or less) without any clear family history [14]. There has been an analysis about association between physical inactivity and CRC development [15]. Obesity is also considered as an independent risk factor for CRC development [16]. Our large prospective study has shown the significant association between high BMI and presence of NAFLD [11].

However, patients with NAFLD harbored a twofold increased risk of colon polyp, regardless of the histopathology findings. In comparison, other studies reported an increased risk of adenomatous polyp, with an OR ranging from 1.28 in patients with NAFLD [7] to 4.89 in patients with NASH, using multivariate analyses [6]. A recent meta-analysis demonstrated that NAFLD was associated with a high risk of colorectal adenoma and the number of polyps, but not with its location, size, and advanced nature [17]. Furthermore, a recent study found that advanced liver fibrosis significantly increases the risk of colorectal adenoma, advanced adenoma, and multiple adenomas [18].

In this study, colon polyps were found in 44.1% of NAFLD patients. Based on pathology point of view, adenoma (tubular and tubulovillous) and serrated adenoma were found in 50% of the patients with polyp. However, only 26.5% NAFLD patients had an adenomatous polyp. It is well known that adenomatous polyp is the most important polyp for CRC development. However, conventional adenomas (tubular, villous, and tubulovillous) are not the only precursor lesions of CRC. It is now recognized that CRC is not a single disease but more represents a constellation of heterogeneous subtypes that develop from different pathways [19]. Interestingly, our study also revealed early malignant transformation of the colon polyp in three NAFLD patients. The current WHO classification lists several precursor lesions of CRC, that is, adenomas, serrated lesions, chronic inflammatory bowed disease, and hamartomatous polyps (juvenile and Peutz-Jeghers polyps) [20]. Serrated lesions are a heterogeneous group that includes hyperplastic polyp, sessile serrated adenoma, and traditional serrated adenoma [21]. The earliest lesion in this group is nondysplastic aberrant crypt foci, which are considered as the precursor lesions of hyperplastic polyps [22]. Inflammatory polyps are generally nonneoplastic and are often related to inflammatory bowed disease or ischemic colitis [23]. However, we still included these polyps since their association with NAFLD has not been well established.

Since NAFLD and colon polyps share similar risk factors, patients with NAFLD might be targeted for CRC screening. Although most colon polyps are usually found in older age and elderly patients, about 25% of patients under 50 years old in our study presented with colon polyps. Considering that regular screening colonoscopy has been recommended for people aged 50 years and older [24], this finding raises the big question of whether colonoscopy should be done earlier, especially in patients with NAFLD. The recent guidelines from the American Cancer Society recommend initiating screening for CRC at the age of 45 years for all average-risk adults [25]. The recommendation to lower the starting age of screening is based on limited empirical data related to outcomes in average-risk individuals in this age group since the previous recommendation to screen at 50 years old has been largely based on expert opinion. However, the average-risk individuals were meant for persons without a history of adenomatous polyp or colorectal cancer or a family tendency of colorectal cancer.

This study has limitations due to retrospective database study. First, it still cannot establish a cause-and-effect relationship and cannot confirm that NAFLD is an independent risk factor of colorectal adenoma. However, these findings have given a new insight into the prevention of CRC development in NAFLD patients. Second, the metabolic risk factors are not analyzed in this study. However, it is already well known that NAFLD is strongly related to metabolic risk factors. A larger and more comprehensive study addressing metabolic factors and colorectal polyps' pathology is needed to find stronger recommendation about screening colonoscopy in every NAFLD patients in clinical practice.

## 5. Conclusions

NAFLD is associated with increased risk of any colorectal polyp, regardless of the histopathological type, compared with patients without NAFLD. This finding implies the necessity to perform screening colonoscopy in patients with NAFLD. Further study is needed to assess other risk factors and patients' eligibility to undergo full colonoscopy when NAFLD is found during routine medical check-up.

## Figures and Tables

**Table 1:** Characteristics of the study subjects (*N* = 138).

Variable	*n* (%)
Age group	
<50 years	43 (31.2)
>50 years	95 (68.8)

Gender	
Male	72 (52.2)
Female	66 (47.8)

Presence of NAFLD	
Yes	68 (49.3)
No	70 (50.7)

Degree of NAFLD (*n* = 68)	
Mild	62 (91.2)
Moderate	5 (7.3)
Severe	1 (1.5)

Presence of polyp	
Yes	49 (35.5)
No	89 (64.5)

**Table 2:** Characteristics of the polyps (*n* = 49).

Characteristic	*n* (%)
Side of polyp (*n* = 49)	
Right	14 (28.6)
Left	35 (71.4)

Histopathology of polyp (*n* = 49)	
Tubular adenoma	21 (42.9)
Inflammatory polyp	7 (14.3)
Serrated adenoma	4 (8.2)
Tubulovillous adenoma	3 (6.1)
Hyperplastic polyp	2 (4.1)
Adenocarcinoma	6 (12.2)
Not biopsied	6 (12.2)

Grade of dysplasia (*n* = 34)	
Mild	16 (47.1)
Moderate	10 (7.2)
High	2 (1.4)
Malignant	6 (4.3)

**Table 3:** Associations among clinical variables and colon polyps (*n* = 138).

Variable	Polyp (+)	Polyp (−)	*p* value	OR	95% CI
Gender					
Male	27 (37.5)	45 (62.5)	0.609	1.200	0.596–2.416
Female	22 (33.3)	44 (66.7)			

Age group					
≤50 years	13 (30.2)	30 (69.8)	0.384	0.710	0.328–1.536
>50 years	36 (37.9)	59 (62.1)			

NAFLD					
Yes	30 (44.1)	38 (55.9)	**0.037**	**2.119**	**1.040–4.318**
No	19 (27.1)	51 (72.9)			

**Table 4:** Association between NAFLD and polyp type (*n* = 43).

NAFLD	Adenoma	Nonadenoma	*p* value	OR	95% CI
Yes	18 (75.0)	6 (25.0)	0.708^*∗*^	0.563	0.120–2.626
No	16 (84.2)	3 (15.8)			

^*∗*^Fisher's exact test.

**Table 5:** Association between NAFLD and polyps' location (*n* = 49).

NAFLD	Right-sided polyp	Left-sided polyp	*p* value	OR	95% CI
Yes	10 (33.3)	20 (66.7)	0.354	1.875	0.491–7.153
No	4 (21.1)	15 (78.9)			

**Table 6:** Association between NAFLD and degree of adenoma dysplasia (*n* = 34).

NAFLD	Mild dysplasia	Higher dysplasia^*∗*^	*p* value	OR	95% CI
Yes	12 (66.7)	6 (33.3)	0.089	3.333	0.815–13.637
No	6 (37.5)	10 (62.5)			

^*∗*^Including moderate dysplasia, severe dysplasia, and malignant carcinoma.

## Data Availability

The database belongs to the Endoscopy Unit of Medistra Hospital, Jakarta.
